# A study of the effect of population aggregation on common prosperity: Evidence from 283 Chinese cities

**DOI:** 10.1371/journal.pone.0292265

**Published:** 2023-10-12

**Authors:** Jia Chen, Ying Ping, Jiefei Jia, Guangliang Li

**Affiliations:** School of Economics and Management, Shanghai Ocean University, Shanghai, China; National University of Sciences and Technology, PAKISTAN

## Abstract

More than 40 years of urbanization in China has brought rapid economic growth, but the uneven development of region and how to achieve common prosperity through urbanization remain a serious concern. This paper analyzes the effect of the urbanization mode of population aggregation in central cities on common prosperity in China. Using panel data of 283 cities from 2004 to 2019, the study constructs a common prosperity index based on the coupling coordination degree of economy, ecology, and society. The Spatial Durbin Model is used to analyze the influence of population aggregation in central cities on common prosperity and the moderating effect of financial self-sufficiency rate. The results show that population aggregation in central cities has a positive effect on common prosperity. There is also a spatial spillover effect, but the impact exhibits an inverted U-shaped characteristic. Moreover, the fiscal self-sufficiency rate has a negative moderating impact on the effect of population aggregation on common prosperity in the early stage, but a positive moderating impact in the later period. This paper concludes by suggesting that the government should promote urbanization, control the size of large cities, accelerate the reform of household registration, and pay attention to the coordinated development of economy, society, and ecology to promote the realization of common prosperity.

## 1 Introduction

**Fig 1:**
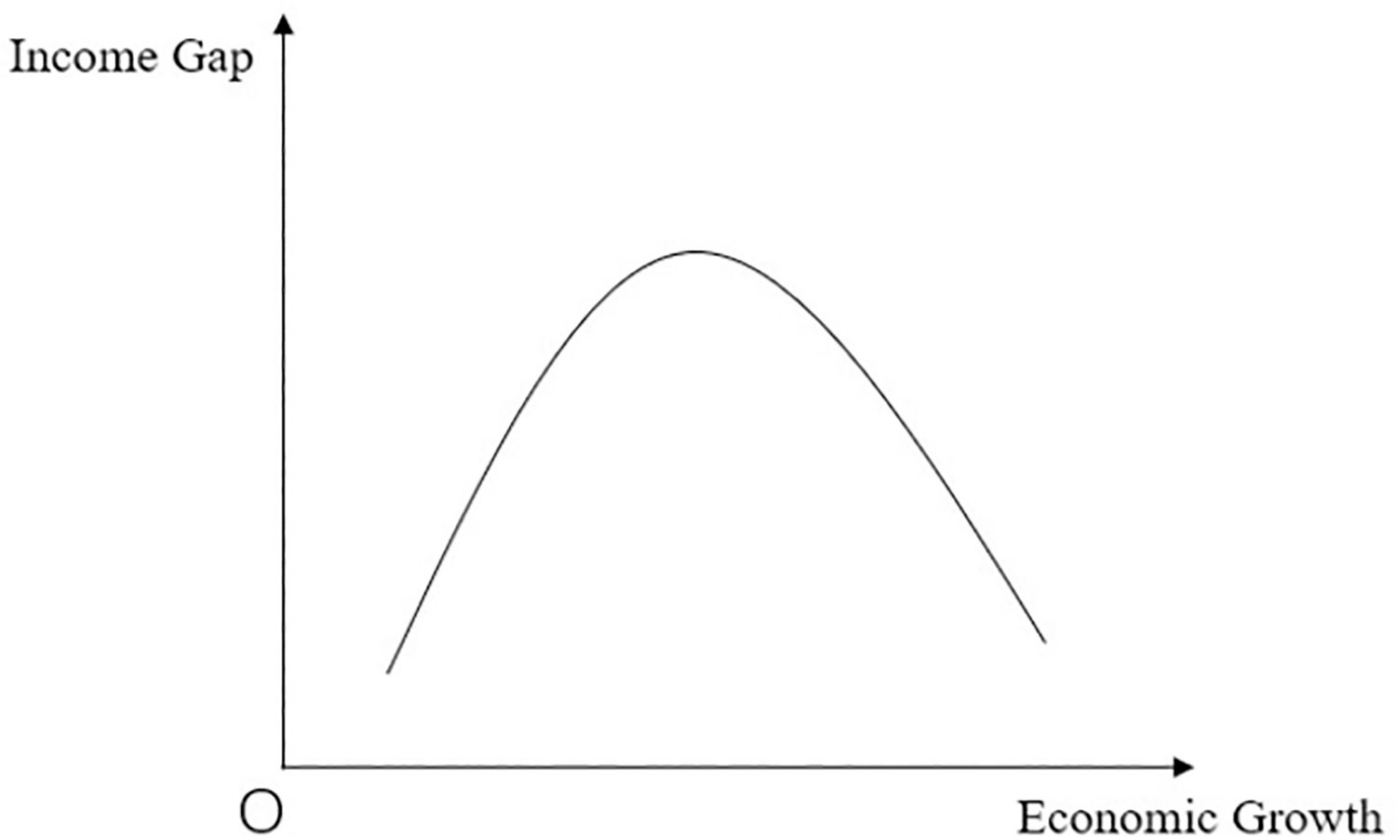
Relationship between economic growth and income gap.

Over the past 40 years, China’s urbanization process has added 730 million urban residents, which has led to a steady rise in living standards for the population as a whole, especially for the approximately 300 million rural migrant workers. In 2020, China achieved a great victory in the battle against poverty [1,2],and the moderately prosperous society has been comprehensively established. According to Lewis’ Theory of Urban Rural Dual Structure, with the deepening of urbanization, Economy was developing, we will achieve balanced development among regions and narrow the gap between the rich and the poor, and eventually achieve common prosperity. Kuznets curve also shown, with developing of economy, we will narrow income gap (As Fig 1). However, the reality is that the uneven development of region remains a serious concern. The gap between rich and poor in developing countries like Chile and Brazil has evolved into the middle-income trap that holds back national development. Then we need to explore how to make urbanization play the role of common prosperity.

The primary contradiction in Chinese society has transformed into the contradiction between the people’s growing needs for a better life and the unbalanced and inadequate development, which determines that promoting common prosperity must be based on economic development. Promoting common prosperity in high-quality growth is needed, which means achieving both material and spiritual prosperity for the people [3–5]. Urbanization is a necessary path for achieving high-quality economic growth and promoting common prosperity. According to the neoclassical theory of economic growth, free movement of factors and specialization are the sources of economic growth. Urbanization promotes the free flow of labor and capital to stimulate economic growth. The factors’ flow to more efficient and developed regions undoubtedly promotes the increase in social and economic efficiency. Based on the theory of comparative advantage, aggregation of factors in areas with high economic efficiency will generate economies of scale, forming economic growth poles, promoting further improvement of socio-economic efficiency, and achieving high-quality economic growth. Meanwhile, it brings advanced technology, capital, and highly skilled personnel to underdeveloped regions by flow of factors, promoting the development of these regions and rural areas in a self-sustaining way [6], which narrows the economic gap between regions. However, the aggregation of factors from underdeveloped regions to developed regions exacerbates the widening economic gap [7]. According to the Chinese Household Finance Survey database(CHFS) in 2017, the top 1% of high-income households account for 69.1% of China’s total household savings, while 95% of households account for only 0.3% of China’s total household savings. Additionally, there are both siphon effects and spillover effects in the process of urbanization [8]. And how to better leverage the spillover effects of urbanization and promote common prosperity is crucial.

Therefore, this study scientifically measures the indicator of common prosperity, analyzes the relationship between common prosperity and population aggregation in central cities, which is a mode of urbanization, and explores the impact mechanism of the urbanization mode of population aggregation in central cities on common prosperity, finally provides a reference for the realization of common prosperity.

This paper proceeds as follows. Section 2 provides a summary of the existing literature on the impact of the urbanization mode of population aggregation in central cities on common prosperity. Section 3 outlines the impact mechanism of population aggregation on common prosperity and the moderating effect of government finance. Section 4 discusses the selection of research indicators, data sources, and research models. Section 5 presents an analysis of the measurement results. Section 6 investigates the moderating effect of fiscal self-sufficiency ratio. Finally, Section 7 summarizes the key findings of this study and offers policy recommendations.

## 2 Literature reviews

**Fig 2:**
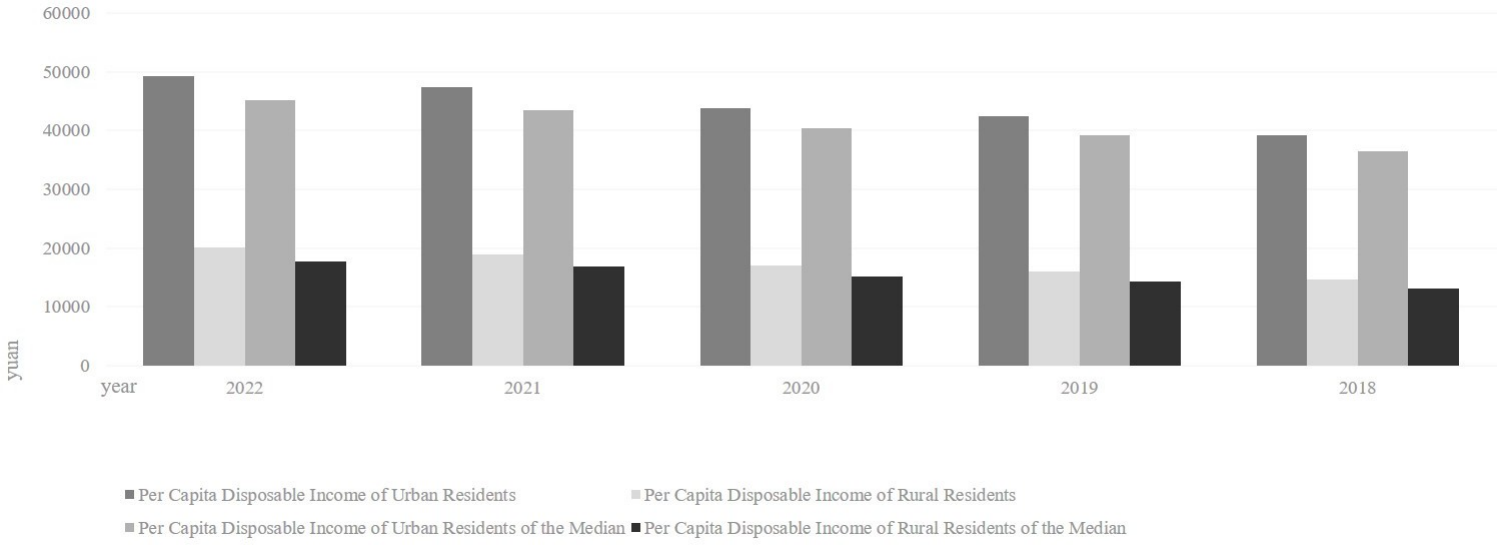
Median and Per capita disposable income of urban and rural residents from 2018 to 2022 (unit: Yuan).

In recent years, scholars have gradually paid attention to the topic of common prosperity. Several scholars have studied the intrinsic connection and theoretical logic between urbanization, city, and common prosperity. They believe that cities are the growth pole of China’s economy. Only through the modernization of cities can we build the modernization of rural areas and achieve overall common prosperity [9].The National Development and Reform Commission announced in 2021 that the urbanization rate of China’s permanent population is 64.72%, which means there are still about 500 million rural permanent residents. According to the 2022 China Statistical Yearbook, the annual disposable income of urban residents is about 2.5 times that of rural residents, as shown in Fig 2. It is impossible to raise the disposable income of the 500 million rural residents in China, a developing country, to the level of urban residents solely by rural industries and social security spending [10]. Moreover, according to the theory of returns to scale, the flow of surplus rural labor to cities will enable them to achieve higher production efficiency and labor remuneration. Meanwhile, the loss of surplus rural labor increases the return of other production factors in rural areas [11]. Thus, it’s necessary to tap the potential of urbanization to achieve common prosperity [12].

Other scholars have explored the path to achieve common prosperity through urbanization, such as promoting new urbanization [13], financial development [14], regional integration [15], digital economy [16,17], evolution of government and market [18], and urban education poverty alleviation [19]. The core of urbanization is population urbanization. Therefore, the key lies in the way in which the population joins urbanization to better achieve its role in common prosperity. This paper focuses on the role of population aggregation in central cities, which is a way of urbanization, in achieving common prosperity. However, current research on population aggregation in central cities mainly focuses on its impact on economic development [20], rural development and environmental health [21], urban transport energy consumption [22], agricultural production factor [23], and narrowing economic disparity [24]. Additionally, the central city strategy has received a lot of criticism, particularly with regard to concerns about siphon effects. This view holds that the development of central cities is achieved at the cost of siphoning off the human, material, and financial resources of other cities [25,26].

Previous literature on population aggregation in central cities lacks attention to common prosperity. Whether further population aggregation in central cities will promote or hinder the common prosperity, and the potential spillover effects or siphon effects to neighboring cities. existing research on the impact of population aggregation in central cities on common prosperity did not form a consistent conclusion. Existing research rarely consider co-prosperity as a the economic-social-ecological system and the moderating effect of fiscal self-sufficiency.

The main contributions of this paper are as follows: First, we selects the coupled coordination of the social subsystem, economic subsystem, and ecological subsystem as the common prosperity indicator, and measures the effect of population aggregation in central cities on common prosperity from the perspective of high-quality growth. Second, by using the spatial econometric model and panel data of 283 prefecture-level cities across China from 2004 to 2019, this study quantitatively analyzes the direct effects and spatial spillover effects of urban resident population aggregation on common prosperity. Furthermore, this paper investigates the moderating impact of fiscal self-sufficiency on the effect of population aggregation on common prosperity in central cities. We hope to better exploit the common prosperity effect of urbanization in China. It provides the decision basis for related policies.

## 3 Theoretical basis and research hypothesis

### 3.1 The impact mechanism of population aggregation on common prosperity

**Fig 3:**
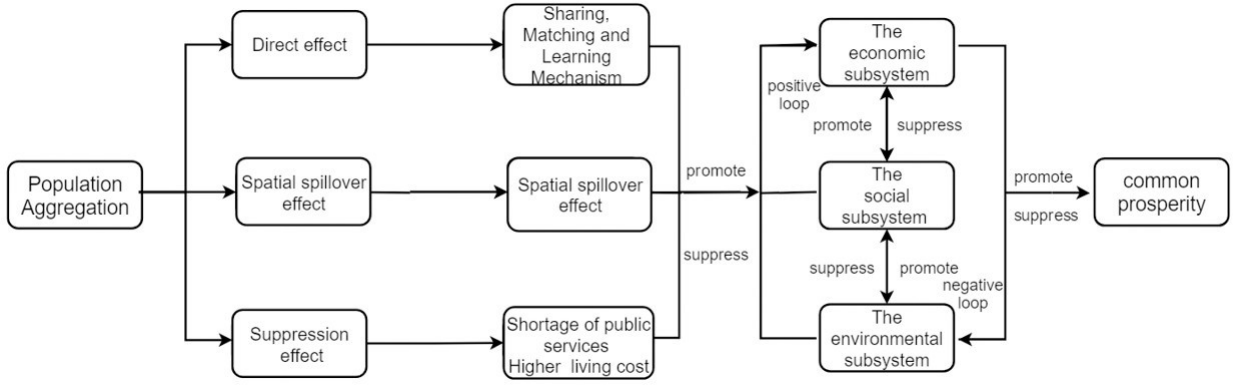
Mechanism of the influence of population aggregation on common prosperity.

The mechanism of the influence of population aggregation on common prosperity is shown in Fig 3.

#### 3.1.1 The internal mechanism of common prosperity

Common prosperity is a systematic concept that includes economic subsystem, social subsystem, and ecological subsystem. The economic subsystem aims at material production and provides products, employment opportunities, and fiscal revenue for economic development. The social subsystem aims at social governance and provides a series of public services for the whole society, such as transportation facilities, educational resources, medical resources, social security, etc. The ecological subsystem aims at ecological civilization construction and provides basic guarantees for ecological revitalization through pollution control and emissions reduction.

The three major subsystems form the common prosperity system, which essentially involves cities formulating policies that match their own development based on internal conditions and external environment. The internal mechanism of the system is that economic growth and income increase bring the requirement of better social governance capabilities, better quality and equal supply of public services, and a livable ecological environment. The government uses fiscal appropriations to improve social governance, public services, and ecological environment. Good social service supply, social governance capabilities, and ecological environment will attract high-quality talents and resources, further promote economic development and increase residents’ income levels. A positive cycle of economic growth, social development, and ecological optimization enriches people’s material and spiritual lives, and fulfills their desires for a better life. Conversely, a negative cycle may impede urban development and lead to a deterioration in the level of common prosperity [27].

#### 3.1.2 The direct effect of population aggregation

Duranton and Puga (2004) summarized the micro-dynamics of population aggregation in central cities into three mechanisms: sharing, matching, and learning [28].

Sharing: Romer (1986) believed that communication generates knowledge [28,29]. Population aggregation in adjacent spaces allows individuals to overcome spatial and temporal barriers of communication, thus facilitating the interaction of workers with different educational backgrounds, ideas, and technologies, promoting the flow and dissemination of knowledge. Meanwhile, the high proximity between urban populations increases learning opportunities and speeds up the learning process for workers, thereby enhancing labor productivity [30]. Second, population aggregation in cities can drive the construction of large-scale infrastructure and provide better public services and educational resources. Conversely, the sharing of public service facilities may improve the efficiency of public services.

Matching: First, population aggregation leads to an abundance of choices in the labor market, with more heterogeneous supply of labor with different preferences and skills, resulting in more suitable matches between workers and enterprises, which may stimulate productivity for both sides. Additionally, the internal demand generated by population aggregation creates economies of scale, promoting competition among firms and contributing to urban productivity improvement [31]. Second, due to the large supply of labor, public service and ecology protection industries can also match appropriate labor force for the corresponding positions, enhancing social governance and ecological governance efficiency [32].

Learning: First, population aggregation produces a learning effect. Due to the intense competition, workers continually enhance their learning of skills, resulting in the "learning by doing" effect. Second, population aggregation attracts a large number of high-quality vocational education resources and skills training, which make knowledge dissemination easier and may improve urban productivity.

In conclusion, population aggregation in central cities can bring about a virtuous cycle of "economic growth—social development—ecological improvement", promoting common prosperity. However, the bearing capacity of the environment is limited. When the degree of population aggregation exceeds the bearing capacity of the city, or when one of the subsystems does not match the development of other subsystems, crowding effects may occur. Further population aggregation will lead to shortage of public services, intense competition, and environmental pollution, resulting in increased cost of living, lower living standards, and widening income disparities, hindering economic development, and forming a vicious cycle, obstructing the common prosperity of the city [33]. Therefore, the following hypothesis is proposed.

Hypothesis 1a: Population aggregation in central cities has a positive impact on the common prosperity of the city, but the impact exhibits an inverted U-shaped characteristic.

Due to the restrictions of China’s household registration system, there is heterogeneity between the urban population and the registered population. Although non-registered residents (mostly migrant workers) live in the same city as registered residents, they cannot enjoy the same treatment. Household registration discrimination may result in non-registered residents not receiving equal treatment in employment opportunities, children’s education, social security, etc., leading to their aggregation in low-level jobs and living in areas with poor environmental conditions such as urban villages [34]. Environmental and social public services, social security, and social governance improve slowly either [35]. There is a significant difference between the wages and conditions of registered and non-registered workers, severely restricting the play of population aggregation effects, affecting the efficiency of resource allocation, and causing the social and ecological systems to lag behind economic development, resulting in the aggregation of non-registered residents producing a siphon effect, thus affecting the realization of common prosperity.

The mobility of the non-registered residents is very high, which results in a large number of left-behind individuals. This affects the stability and development of the social system. Furthermore, discrimination against non-registered residents may lead to long-term inequalities in employment and education, causing these individuals to ignore investments in their children’s education and human resources, inhibiting the improvement of labor efficiency, and hindering the development of common prosperity.

Hypothesis 1b: Due to the existence of household registration discrimination, the increasing deviation between non-registered residents and household registered residents has a negative effect on urban common prosperity and generates negative spatial effects.

#### 3.1.3 The spatial spillover effect of population aggregation

Due to the dynamic flow of various factors such as knowledge, technology, and population in space, the central city and surrounding areas may promote the development of a certain advantageous system in the economy, society, and environment of the region. The methods for development includes: economic connections, exchange of governance methods, neighborhood demonstration effects of mutual learning, assistance and mutual assistance of educational resources, talent introduction, industry introduction, external spillover of environmental governance, mobility and communication of individuals. The development of a certain advantageous system leads to a virtuous cycle of "economic growth, social development, ecological improvement", thus transforming the development advantages of a particular system into the overall development of the economy, society, and ecology, and accomplishing common prosperity in a global sense through "the richer bring along the less richer" approach. However, when the population aggregates to the central city to a certain extent, further promotion of aggregation may produce a negative effect on the surrounding cities through the siphon effect.

Hypothesis 2: The spatial spillover effect of population aggregation in central cities on common prosperity in surrounding cities exists and exhibits an inverted U-shaped characteristic. That means, after reaching a certain point, further increase in population aggregation may have a negative effect on the common prosperity of surrounding cities, namely, the siphon effect.

#### 3.1.4 The moderating effect of fiscal self-sufficiency

The concept of fiscal self-sufficiency is commonly used to evaluate the financial soundness and autonomy of local governments. In China’s tax-sharing system, it is measured as the ratio of budgetary fiscal revenue to budgetary fiscal expenditure. Enhancing fiscal self-sufficiency can lead to better control of expenditure, greater efficiency in the use of fiscal funds, and a reduction in the negative effects of excessive local government debt on the economy. It also enables local governments to invest in areas that are most suitable for their actual conditions, thereby increasing future returns on fiscal funds. However, local governments often face pressure to increase expenditure to boost local economies, which may have short-term effects and reduce the efficiency of fund utilization and future development. This often leads to local governments relying on land finance and causing a vicious cycle of rising house prices and increased living costs [36].

In the early stages of urbanization, local governments require significant funds for supporting facilities construction, which can lead to increased expenditures for social and ecological governance. In this context, increasing fiscal self-sufficiency may reduce local government expenditure funds and negatively impact the common prosperity effect of population aggregation. However, in the later stages of population aggregation, due to the large size of the city, it becomes necessary to alleviate the siphon effect caused by urbanization, increase fiscal self-sufficiency, and promote the balanced development of surrounding areas.

Based on the above arguments, this paper proposes the following hypothesis.

Hypothesis 3a: Fiscal self-sufficiency has a positive effect on common prosperity.

Hypothesis 3b: In the primary stage of population aggregation, fiscal self-sufficiency is expected to have a negative moderating effect on the common prosperity effect, while in the subsequent stage of population aggregation, it is expected to have a positive moderating effect on the common prosperity effect of population aggregation.

## 4 Research design

### 4.1 Data processing

#### 4.1.1 Data source and indicator description

The data used in this paper mainly comes from China Urban Statistical Yearbook, China Statistical Yearbook, and the National Bureau of Statistics. Missing data is supplemented using the moving average method to fill in gaps from 4 years before and after. Following the method of Li (2022) [27], this paper selects 40 indicators to construct the indicator systems of economic subsystem, social subsystem, and ecological subsystem. Using the coupling coordination degree measurement for common prosperity, this paper traces the development of 283 cities from 2004 to 2019, conducts spatio-temporal analysis, and explores the role of population aggregation in common prosperity.

The specific indicators of explained variables (s*core*_*cou*r_) are constructed as follows.

Indicators of economic subsystem: GDP, per capita GDP, and GDP growth rate measure economic growth [37]; value-added of the primary, secondary, and tertiary industries, and the proportion of the primary, secondary, and tertiary industries to GDP measure economic structure; the number of domestic enterprises and the total industrial output value of domestic enterprises measure the development of domestic enterprises; the year-end balance of deposits and loans of financial institutions measure capital flow; the actual amount of foreign investment used in that year measures the business environment; and the total retail sales of consumer goods measure residents’ consumption ability.

Indicators of social subsystem: The number of employees in social public management and social organizations and the total fixed asset investment measure the human and financial resources of public management [38]; the number of public libraries and the total number of books, as well as the number of libraries per 100 people, measure the construction of public library resources; the number of hospitals, health centers, and health institutions, as well as the number of hospital beds and doctors, measure the supply of medical resources; the amount of freight and passenger transportation measures the transportation situation; and education expenditure, the number of general institutions of higher learning, and the number of students in general institutions of higher learning measure the education resources situation.

Indicators of ecological system: Production, removal, and emission of industrial sulfur dioxide, industrial soot removal and emissions measure air pollution and treatment of air pollution; industrial wastewater discharge and the amount of industrial wastewater discharge meeting standards measure the degree of water pollution and management [38]; the centralized treatment rate of pollution treatment plants, the rate of domestic sewage treatment, and the rate of harmless treatment of domestic garbage measure ecological management.

The explanatory variable is population aggregation [39].

The control variables are: The deviation rate of Financial self-sufficiency rate, the deviation rate of urban registered population and permanent population, the degree of marketization, the year-end employment rate per unit, the number of employees in scientific research, health, social insurance, and social welfare, and the number of employees in the electricity, gas, and water production and supply industry as control variables corresponding to the three major systems of economy, society, and ecology, respectively.

This paper eliminates the influence caused by the dimension of the indicator using normalization. Based on the characteristics of the selected variables, positive extreme value normalization is used.

#### 4.1.2 Setting of Indicator weights

By normalizing the processed variables v_i_ and the weights constructed by the entropy weighting method w_i_, the score of each system for each prefecture-level city is calculated as: $\mathrm{score}=\sum_{i=1}^{n}w_{i}v_{i}$, and the coupling degree index is shown in Eq (1).


$$score_{cou}={\left[\frac{score_{econ}+score_{soc}+score_{envir}}{{\left(score_{econ}+score_{soc}+score_{envir}\right)}^{3}}\right]}^{\frac{1}{3}}$$ (1)

The overall coupling coordination degree index is shown in Eq (2).


$$score_{cour}=\sqrt{score_{cou}+(w_{1}score_{econ}+w_{2}score_{soc}+w_{3}score_{envir})}$$ (2)

This paper holds that economic development, social system, and ecological protection are equally important in the coordinated development process, so the weights of the three systems are set to be equal: w_1_ = w_2_ = w_3_

The descriptive statistics of all variables are shown in Table 1.

**Table 1:** Description of variables.

Variable	Symbols	Number of observations	Average value	Standard deviation	Minimum value	Maximum value	Measurements
Degree of common prosperity	SCOURcour	4528	0.4196	0.0402	0.3337	0.5796	
Population Aggregation	PA	4528	0.0060	0.0037	0.0003	0.0220	Using the ratio of the resident population of the city in that year to the total urban resident population of the country in that year
Financial self-sufficiency rate	FSR	4528	0.4694	0.2259	0.0544	1.5413	Using local fiscal general budget revenue over local fiscal general budget expenditure
Deviation rate of urban household registration from the resident population	PDR	4528	0.1196	0.2699	0.0000	3.8495	Ratio of resident population to household population minus one in absolute terms
Scientific expenditure	SE	4528	0.0096	0.0345	0.0000	1.0000	The science expenditure item in the Urban Statistics Yearbook is positively normalized
Number of persons employed in the health, social security and social welfare sector	NOEW	4528	0.0989	0.0840	0.0000	1.0000	The number of persons employed in the health, social security and social welfare sector in the Urban Statistics Yearbook is normalized
Level of marketability	ML	4528	0.1054	0.0834	0.0028	0.9170	Number of urban private and self-employed persons compared to resident population
Year-end unit employment rate	NOEYR	4528	0.1079	0.0653	0.0280	0.6725	Number of persons employed in units at the end of the year in the Urban Statistical Yearbook / Number of permanent urban residents at the end of the year
Number of persons employed in the electricity, gas and water production and supply industry	NOEE	4528	0.0877	0.0739	0.0000	1.0000	The number of persons employed in the electricity, gas and water production and supply industry in the Urban Statistics Yearbook has been positively normalized
Secondary term for population aggregation	PA^2	4528	0.0000	0.0001	0.0000	0.0005	Secondary term for population aggregation

### 4.2 Spatial autocorrelation test

To use spatial econometric models, it is necessary to test the spatial autocorrelation effect of variables and measure whether there are spatial impacts. The test results are shown in the Table 2.

**Table 2:** Spatial correlation test for common wealth coupling in 283 cities across the country, 2004–2019.

	2004	2005	2006	2007	2008	2009	2010	2011
Moran’s I	0.240	0.273	0.266	0.272	0.294	0.276	0.271	0.248
P-value	0.005	0.000	0.000	0.000	0.000	0.001	0.000	0.001
	2012	2013	2014	2015	2016	2017	2018	2019
Moran’s I	0.248	0.246	0.250	0.263	0.274	0.279	0.275	0.269
P-value	0.001	0.001	0.003	0.000	0.000	0.000	0.000	0.001

It can be seen that the overall Moran’s I coefficients are all positive, and the results are significant. Therefore, it can be concluded that there is a spatial positive correlation effect.

**Fig 4:**
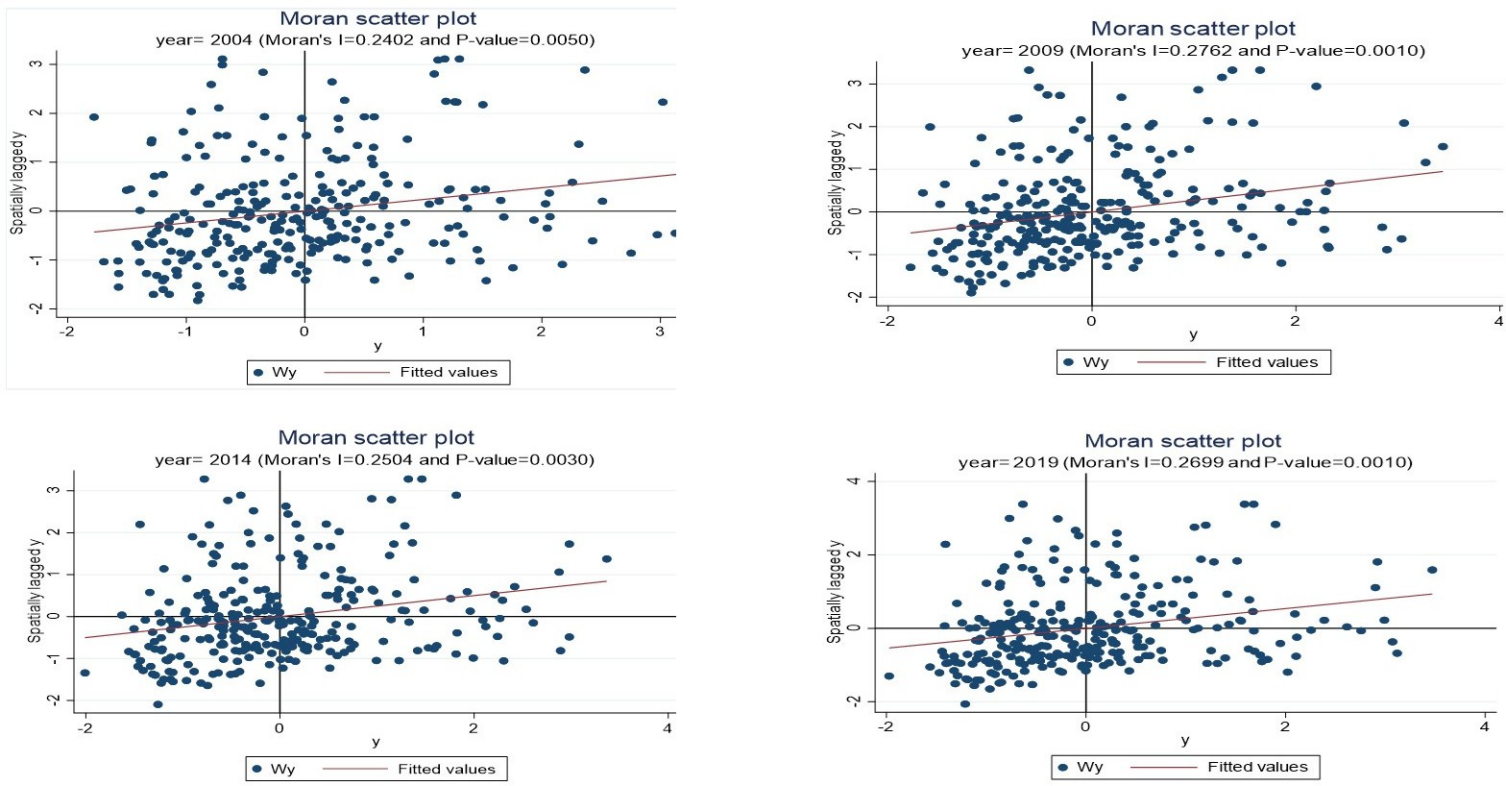
Moran scatter plot in 2004 (top left) 2009 (top right) 2014 (bottom left) and 2019 (bottom right).

We draw the local Moran scatter plot in order to observe the regional correlation and aggregation of common affluence in different cities intuitively. The results are shown in Fig 4. In this paper, the local spatial scatter plots of 2004, 2009,2014 and 2019 are drawn to further illustrate the existence of obvious spatial aggregation effect, which once again verifies the necessity of considering spatial effect.

### 4.3 Model specification

#### 4.3.1 The basic models of spatial model

Generally, traditional spatial econometric models can be divided into three main categories [40]. The first category is the Spatial Lag Model (SLM), which emphasizes the interdependence of the dependent variable in space. That means, the dependent variable may affect other regions through spatial interaction. The second category is the Spatial Error Model (SEM), whose basic assumption is that the spatial spillover effect is mainly transmitted through the spatial error term. The third category is the Spatial Durbin Model (SDM), which considers the spatial correlation of all explanatory variables and the dependent variable simultaneously.

The basic models of Spatial Lag Model (Formula 3), Spatial Error Model (Formula 4), and Spatial Durbin Model (Formula 5) are shown as follows:

$$y_{n}=a+\rho \omega_{n}y_{n}+\beta_{1}x_{n}+\varepsilon_{n}$$ (3)

$$y_{n}=a+\beta_{1}x_{n}+u_{n},u_{n}=\delta \omega_{n}u_{n}+\varepsilon_{n}$$ (4)

$$y_{n}=a+\rho \omega_{n}y_{n}+\beta_{1}x_{n}+\beta_{2}\omega_{n}x_{n}+\varepsilon_{n}$$ (5)

Where *y*_*n*_ represents the explained variable, *x*_*n*_ is the explanatory variable, *ω*_*n*_ is the spatial weight matrix, and *ρ* is the adjacent area of *ω*_*n*_*y*_*n*_ for the dependent variable *y*_*n*_. And *β*_1_ is the coefficient of influence of *x*_*n*_ on *y*_*n*_. *β*_2_ is the coefficient of influence of *ω*_*n*_*x*_*n*_ on *y*_*n*_ in the adjacent area. *ε*_*n*_ is the stochastic disturbance, which obeys *ε*_*n*_~*N*(0, *σ*^2^*I*_n_).

#### 4.3.2 Spatial weight matrix

The spatial weight matrix is mainly divided into three categories: binary matrix, distance matrix, and economic matrix. The binary matrix is a 0–1 matrix that uses 0 to indicate non-neighboring regions and 1 to indicate neighboring regions, but it cannot reflect the strength of the spatial relationship of different geographic distances. The distance matrix is based on the different spatial distances of individual units and constructs a matrix that is more consistent with the distance attenuation law of spatial interaction. Therefore, the distance matrix used in this paper for spatial correlation analysis and spatial regression results is set as follows (Eq 6):

$$W_{ij}(d)=\{\begin{array}{l} \frac{1}{d_{ij}^{2}},i\neq j\\ 0,i=j \end{array}\}$$ (6)

Where *W*_*ij*_ represents the spatial weight between city i and city j, and $d_{\mathrm{ij}}^{2}$ represents the squared Euclidean distance between city i and city j. Although the distance matrix can reflect the distance attenuation law of spatial interaction, it ignores the influence of economic relationships. Therefore, the economic geographic weight matrix is selected as a robustness test in this paper, and the matrix is as follows (Eq 7):

$$W_{ij}(d)=\{\begin{array}{l} \frac{\left|{\bar{Q}}_{i}-\bar{Q_{j}}\right|}{d_{ij}},i\neq j\\ 0,i=j \end{array}\}$$ (7)

Where $\bar{Q_{i}}$ and $\bar{Q_{j}}$ are the economic weights between city i and city j and the average value of the logarithm of per capita GDP from 2004 to 2019, respectively.

## 5 Empirical results and analysis

The empirical results of the three models are shown in Table 3. Based on the LM test, the effect test, and the Hausman test, all results are significant at the level of 1%, indicating that the individual and spatial fixed effects model should be chosen. Regardless of which model is used to explain the population aggregation (PA) in the regression, the estimated coefficient is very significant at the level of 1%, indicating that the results are robust. However, there are some slight differences in the significance levels, and population aggregation (PA), self-sufficiency rate (FSR), scientific expenditures (SE), the number of employees in health, social insurance, and social welfare industries (NOEW), marketization level (ML), and the number of employees in the electricity, gas, and water production and supply industry (NOEE) all have very significant positive effects, which are economically meaningful and can effectively promote the development of urban common prosperity. The deviation rate between urban household registration and permanent population (PDR) is also very significant, negatively correlated with the degree of common prosperity, which is economically meaningful, and reducing household registration restrictions can promote the development of urban common prosperity.

**Table 3:** Individual, time-dependent double fixed effects models.

	SAR		SEM		SDM	
		W1*		W1*		W1*
*score_cour_*		0.3858***			0.2916***	
PA	2.8539***		2.5394***		2.1889***	8.4635***
FSR	0.0097******		0.0099***		0.0087***	0.0099***
PDR	-0.0042***		-0.0039**		-0.0039**	-0.0112**
SE	0.4579***		0.0468***		0.0444***	0.0106
NOEW	0.0491***		0.0448***		0.0487***	0.0032
ML	0.0047***		0.0048**	-	0.0042**	0.0081
NOEY	0.0039		0.0063**	-	0.0014	-0.0050
NOEE	0.0137***-		0.0131***		0.1513***	-0.0106
PAL^2	-202.0578***		-191.719***		-175.963***	-375.572***
*e.score_cour_*				0.3759***		
*δ*	0.00003***		0.00003***		0.00003***	
Likely hood	17097		17064		17129	
Individual fixed effects	Yes	Yes	Yes	Yes	Yes	Yes
Time fixed effects	Yes	Yes	Yes	Yes	Yes	Yes

Note: All initial data in the table are obtained from the 2004–2019 China Statistical Yearbook of each prefecture-level city.

*, ** and *** denote 10%, 5% and 1% significance levels respectively, the same below.

The year-end unit employment rate (NOEYR) is not significant, which may be due to restrictions on the staffing and state-owned enterprise reforms, which have not resonated with economic development, and therefore have no significant effect on regional common prosperity development. Furthermore, using the Wald and LR tests to discriminate the models, the results of the spatial lag test and spatial error test of the Wald and LR tests (shown in Table 4) are significant at the level of 1%, indicating that the SDM model with individual and time fixed effects is the best model for empirical research in this paper. Therefore, this paper further analyzes the SDM model. The model formula is as follows (Eq 8):

$$\begin{array}{l} score_{cour}=a+\rho \omega_{n}score_{cour}+\beta_{1}PA+\beta_{2}FSR+\beta_{3}PDR+\beta_{4}SE\\ +\beta_{5}NOEW+\beta_{6}ML+\beta_{7}NOEY+\beta_{8}NOEE+\beta_{9}{\mathrm{PA}}^{2}+\theta_{1}\omega_{n}PA\\ +\theta_{2}\omega_{n}FSR+\theta_{3}\omega_{n}PDR+\theta_{4}\omega_{n}SE+\theta_{5}\omega_{n}NOEW+\theta_{6}\omega_{n}ML+\theta_{7}\omega_{n}NOEY+\theta_{8}\omega_{n}NOEE+\theta_{9}\omega_{n}{\mathrm{PA}}^{2}+\varepsilon_{n} \end{array}$$ (8)

**Table 4:** Test of spatial model selection.

Model	W1			W2		
Test method	Tests	Statistics	P-value	Tests	Statistics	P-value
LM-test	LM-spatial lag	233.12	0.008	LM-spatial lag	219.51	0.000
	Robust LM-spatial lag	157.42	0.000	Robust LM-spatial lag	30.025	0.000
	LM-spatial error	9591.36	0.000	LM-spatial error	398.03	0.000
	Robust LM-spatial error	9515.65	0.003	Robust LM-spatial error	208.54	0.000
LR-test	LR-spatial lag	26.58	0.000	LR-spatial lag	44.23	0.0000
	LR-spatial error	39.21	0.0000	LR-spatial error	76.41	0.0000
Wald-test	Wald-spatial lag	63.06	0.000	Wald-spatial lag	44.30	0.0000
	Wald-spatial error	130.50	0.0000	Wald-spatial error	76.92	0.0000

Table 5 presents the partial derivative decomposition of the SDM model to further analyze the spatial effects and examine the direct, indirect, and total effects of the variables. The results of partial derivative decomposition mainly focus on the geographic distance matrix W1 and the economic geographic distance matrix W2. The direct effect of population aggregation (PA) is significantly positive at the level of 1%, indicating that further population aggregation in central cities may lead to learning, matching, and sharing effects, promote economic development, and drive the improvement of social governance capabilities and ecological governance. The direct effect is greater than the crowding effect, achieving a virtuous cycle of "economic growth—social development—better ecology". The spatial effect and total effect are both significantly positive at the level of 1%, indicating that the increase in population aggregation produces positive spatial spillover effects. The possible reason is that measures such as knowledge exchange, talent exchange, and industrial transfer from the central city spill over to surrounding cities, producing "the richer bring along the less richer" effect and promoting the development of common prosperity in surrounding cities. The direct and total effect coefficients of the quadratic term of population aggregation (PA^2) are negative at the level of 1%, indicating that the impact of population aggregation on urban common prosperity increases first and then decreases. The spatial effect is also negative, indicating that when the degree of population aggregation is very high, with the further increase in population aggregation, the spillover effect on common prosperity of surrounding cities becomes negative, turning from a spillover effect to a siphon effect, which is not conducive to the common prosperity of surrounding cities. The positive and significant coefficients of the self-sufficiency rate of political entities (FSR), scientific expenditure (SE), the number of employees in health, social insurance and social welfare industries (NOEW), marketization level (ML), and the number of employees in electricity, gas, and water production and supply industries (NOEE) all demonstrate positive effects on urban common prosperity, which is consistent with the economic logic. The negative and significant coefficients of the deviation rate between urban household registration and permanent population (PDR) indicate a negative correlation with common prosperity, which is in line with economic logic. Relaxing household registration restrictions can promote the development of common prosperity in cities. The year-end unit employment rate (NOEYR) is not significant, possibly due to restrictions on the number of employed units caused by institutional arrangements and state-owned enterprise reforms, which does not resonate with economic development and therefore has little effect on regional common prosperity. In summary, hypothesis 1a, hypothesis 1b and hypothesis 2 are proven.

**Table 5:** Decomposition of spatial spillover effects based on the spatial Durbin model.

		SDM			SDM	
		W1			W2	
Variables	Direct effects	Indirect effects	Total effect	Direct effects	Indirect effects	Total effect
PA	2.4730***	12.6531***	15.1262***	3.1336***	2.6324***	5.7661***
	(0.5001)	(2.0228)	(1.9752)	(0.5065)	(0.5856)	(0.7063)
FSR	0.0089***	0.0172***	0.0262***	0.0102***	0.0037***	0.0139***
	(0.0012)	(0.0041)	(0.0039)	(0.0012)	(0.0013)	(0.0016)
PDR	-0.0040***	-0.0167*	-0.0207***	-0.0034**	-0.0022	-0.0056**
	(0.0015)	(0.0072)	(0.0075)	(0.0015)	(0.0020)	(0.0025)
SE	0.0452***	0.0317	0.0770***	0.0491***	0.0151***	0.0643***
	(0.0046)	(0.0177)	(0.0186)	(0.0046)	(0.0058)	(0.0073)
NOEW	0.0491***	0.0251	0.0742***	0.0461***	-0.0059	0.0402***
	(0.0041)	(0.0184)	(0.0192)	(0.0042)	(0.0046)	(0.0065)
ML	0.0046**	0.0125*	0.0171**	0.0056***	0.0021	0.0077***
	(0.0019)	(0.0071)	(0.0071)	(0.0019)	(0.0021)	(0.0027)
NOEYR	0.0013	-0.0061	-0.0047	0.0056**	0.0052*	0.0109**
	(0.0032)	(0.0119)	(0.0120)	(0.0032)	(0.0032)	(0.0045)
NOEE	0.0148***	-0.0078	0.0069	0.0127***	0.0010	0.0137***
	(0.0022)	0.0099	(0.0104)	(0.0022)	(0.0022)	(0.0033)
PA^2	-188.33***	-593.23***	-781.56***	-218.85***	-122.00***	-340.86***
	(18.218)	(82.145)	(81.284)	(18.392)	(24.600)	(29.582)
Individual fixed effects	Yes	Yes	Yes	Yes	Yes	Yes
Time fixed effects	Yes	Yes	Yes	Yes	Yes	Yes

## 6 Further analysis of the moderating effect of fiscal self-sufficiency ratio

Previous research has confirmed that population aggregation and fiscal self-sufficiency ratio not only have a positive impact on common prosperity in central cities but also have spillover effects in neighboring areas. This paper will further empirically analyze the moderating effect of fiscal self-sufficiency ratio on the impact of population aggregation on common prosperity from the perspective of coordinated interaction. Does the fiscal self-sufficiency ratio promote or hinder the common prosperity effect when the population aggregation increases?

### 6.1 Spatial moderating effect

This paper takes the moderating effect of fiscal self-sufficiency ratio on the process of population aggregation affecting common prosperity into consideration. Therefore, the interaction term (PA*FSR) between fiscal self-sufficiency ratio and population aggregation and the interaction term (PA*FSR) between fiscal self-sufficiency ratio and the squared population aggregation are added to the baseline model, and the model is designed as follows (Eq 9):

$$\begin{array}{l} score_{cour}=a+\rho \omega_{n}score_{cour}+\beta_{1}PA+\beta_{2}FSR+\beta_{3}PDR+\beta_{4}SE+\beta_{5}NOEW+\\ \beta_{6}ML+\beta_{7}NOEY+\beta_{8}NOEE+\beta_{9}{\mathrm{PA}}^{2}+\beta_{10}PA*FSR+\beta_{11}{\mathrm{PA}}^{2}*FSR\\ +\theta_{1}\omega_{n}PA+\theta_{2}\omega_{n}FSR+\theta_{3}\omega_{n}PDR+\theta_{4}\omega_{n}SE+\theta_{5}\omega_{n}NOEW+\theta_{6}\omega_{n}ML\\ +\theta_{7}\omega_{n}NOEY+\theta_{8}\omega_{n}NOEE+\theta_{9}\omega_{n}{\mathrm{PA}}^{2}+\theta_{10}\omega_{n}PA*FSR+\theta_{11}\omega_{n}{\mathrm{PA}}^{2}*FSR+\varepsilon_{n} \end{array}$$ (9)

The spatial effect decomposition is shown in Table 6. The direct effect, spatial effect, and total effect of the interaction term (PA*FSR) under two weight matrices are significantly negative at the level of 1%. Obviously, the fiscal self-sufficiency ratio has a negative impact on the effect of population aggregation on common prosperity. The direct effect, spatial effect, and total effect of the interaction term (PA^2^*FSR) are significantly positive at the level of 1%. The reason is that in the early stage of population aggregation (i.e., the left side of the inverted U-shaped curve), due to the large number of people aggregation in the central city, appropriately expanding fiscal expenditures and increasing capital construction efforts will promote the common prosperity effect of population aggregation. Increasing the fiscal self-sufficiency ratio and controlling expenditures will obviously have a negative moderating effect on the common prosperity effect of population aggregation, showing a substitution effect. When the population aggregation enters the later stage (i.e., the right side of the inverted U-shaped curve), the city has become very large, and overcrowding and siphon effects have occurred due to further population aggregation in the central city, which is not conducive to common prosperity. Increasing the fiscal self-sufficiency ratio can help control the debt scale, increase the city’s financial freedom to use funds, invest more in regions with good development prospects, and delay the overcrowding and siphon effects of the city. In summary, hypotheses 3a and 3b are proven.

**Table 6:** Decomposition of spatial spillover effects with the inclusion of interaction terms.

		SDM			SDM	
		W1			W2	
Variables	Direct effects	Indirect effects	Total effect	Direct effects	Indirect effects	Total effect
PA	4.1395***	16.570***	20.710***	5.6024***	5.3522***	10.954***
	(0.6753)	(2.5076)	(2.5043)	(0.6664)	(0.8384)	(1.0317)
FSR	0.0229***	0.0443***	0.0673***	0.0290***	0.0192***	0.0482***
	(0.0032)	(0.0109)	(0.0112)	(0.0032)	(0.0035)	(0.0046)
PDR	-0.0049***	-0.0194***	-0.0244***	-0.0047**	-0.0034**	-0.0082**
	(0.0015)	(0.0068)	(0.0071)	(0.0015)	(0.0019)	(0.0025)
PA^2	-252.67***	-806.00***	-1058.68**	-328.30***	-264.56***	-592.86***
	(31.702)	(131.057)	(129.747)	(31.105)	(45.048)	(53.484)
PA*FSR	-3.3461***	-9.0679***	-12.414***	-4.9075***	-4.7109***	-9.6184***
	(0.8757)	(3.4859)	(3.5927)	(0.8659)	(1.0452)	(1.3519)
PA^2*FSR	141.513***	533.767***	675.281***	234.685***	264.520***	499.266***
	(53.517)	(236.335)	(243.276)	(53.258)	(67.504)	(87.059)
Control variables	Yes	Yes	Yes	Yes	Yes	Yes
Individual fixed effects	Yes	Yes	Yes	Yes	Yes	Yes
Time fixed effects	Yes	Yes	Yes	Yes	Yes	Yes

## 7 Conclusion and implications

As the task of achieving common prosperity becomes increasingly urgent and important, the relationship between urbanization and common prosperity is crucial, and the population aggregation in central cities is undoubtedly the key factor in urbanization. What’s more, the role of finance in the process of population aggregation in the central city cannot be ignored. Based on the analysis of panel data from 283 prefecture-level cities in China from 2004 to 2019, this study clarifies the impact of population aggregation in central cities on common prosperity, and uses the spatial Durbin model to explore the impact of population aggregation and fiscal self-sufficiency on urban common prosperity. Furthermore, this study analyzes the moderating effect of fiscal self-sufficiency on the impact of population aggregation in central cities on common prosperity. The results show that:

1. The population aggregation in central cities has a significant promoting effect on urban common prosperity, but the impact exhibits an inverted U-shaped characteristic. And there is a positive spatial spillover effect, which also exhibits an inverted U-shaped characteristic.
2. The deviation rate between registered population and permanent population, i.e., the expansion of the permanent non-registered population, has a significant negative impact on common prosperity, and there is also a negative spatial effect. The reason is that the permanent non-registered population has a high degree of mobility, which results in a large number of left-behind phenomena, affecting the stability and development of the social system. Moreover, household registration discrimination may lead to long-term inequality in employment, education, and other aspects, which often makes these people neglect investment in their children’s education and human resources, suppresses the efficiency improvement of labor, and is not conducive to the development of regional common prosperity.
3. The fiscal self-sufficiency ratio has a significant positive impact on common prosperity, and there is a positive spatial spillover effect.
4. There is a significant negative moderating impact on the effect of population aggregation on common prosperity in the early stage (i.e., the left side of the inverted U-shaped curve), because the process of increasing fiscal self-sufficiency may control spending. However, in the later stage of population aggregation in central cities (i.e., the right side of the inverted U-shaped curve), there is a significant positive moderating effect, because at this stage, the crowding effect and siphon effect of population aggregation in the city have already occurred, which is not conducive to common prosperity. Improving fiscal self-sufficiency can help control the size of debt, improve the freedom of urban funds use, invest more in regions with good development prospects, and alleviate the crowding effects and siphon effects of cities.

Based on the above conclusions, the following suggestions are proposed:

1. Adhere to the urbanization strategy and encourage population concentration in large and central cities, while paying attention to moderate urban size to prevent the negative effects of the "big city disease" [41].Some people don’t want to move to large cities and some people cannot settle in large cities, we should encourage them gather in small cities and central counties, as a supplement to the strategy of large cities, forming a "large concentration, small centralization" pattern of urban development to enhance the role of population aggregation in promoting common prosperity [42].
2. The government should accelerate the reform of the household registration system, reduce the deviation between registered population and permanent population, eliminate household registration discrimination, and limit its adverse effects on common prosperity [43].
3. he government should pay attention to the left-behind children and elderly people left behind by non-registered permanent residents, especially migrant workers, provide more financial assistance for their children’s education, and care more about the psychological, health, and medical conditions of the left-behind elderly people.
4. The central government should reasonably and moderately delegate financial power to local governments, improve the financial self-sufficiency of local governments, so that local governments can use financial resources more flexibly in areas more suitable for local development [44]. Local governments should not expand spending and invest in short-term projects, leading to an unbalanced and rising government debt scale that is not conducive to subsequent urban development.
5. overnments at all levels should increase their spending on science, speed up the reform of decentralization, improve the degree of marketization in cities, make the allocation of resources more efficient, and at the same time play a good redistributive role, strive to achieve equalization of the supply of public services, improve the governance capacity, and pay attention to environmental protection [45], leading to a virtuous cycle of "economic growth—social development—better ecology", thus achieving common prosperity of peoples’ material and spiritual life from local to the whole.

## Supporting information

S1 Data (RAR)
